# Longitudinal analysis of social and community factors effective in increasing the number of liver donors in the United States

**DOI:** 10.1097/MD.0000000000039694

**Published:** 2024-09-20

**Authors:** Ender Anilir

**Affiliations:** a Biruni University Hospital Organ Transplantation and Hepatobiliary Surgery Center, İstanbul, Turkey.

**Keywords:** donor, liver, social factors, transplantation

## Abstract

In this research, it was aimed to evaluate of social and community factors effective in increasing the number of liver donors. Descriptive and relational scanning models were used to conduct the research. Data on liver donors was gathered from the USA Health Resources & Services Administration’s Organ Procurement and Transplantation Network between 1988 and 2023. The United States (USA) World Bank Country Reports provided the mortality rates. The data was analyzed using Spearman rho correlation, year-controlled partial correlation, and Generalized Linear Model-Logit analysis. Deceased donor numbers were significantly and negatively correlated with government health expenditure (*r* = −0.816; *P* < .01), current health expenditure (*r* = −0.768; *P* < .01), female education attainment (*r* = −0.804; *P* < .01) and Gini index (*r* = 0.434; *P* < .05). Living donor numbers were significantly and negatively correlated with government health expenditure (*r* = −0.731; *P* < .01), current health expenditure (*r* = −0.781; *P* < .01), male percentage (*r* = −0.786; *P* < .01), female education attainment (*r* = −0.640; *P* < .05), employment (*r* = 0.751; *P* < .01), GDP (*r* = −0.792; *P* < .01) and Gini index (*r* = −0.486; *P* < .01). Living donor numbers were significantly and positively correlated with age dependency (*r* = 0.815; *P* < .01). Generalized Linear Model-Logit (GLM-L) results showed that effect of female education attainment had significant contribution on deceased liver donor (B = −3290.605; *P* < .01). Effects of significantly correlated community factors on living liver donor numbers were found to be statistically insignificant (*P* > .05). Research findings reveal that among community factors, especially women’s participation in education has a statistically significant effect on liver donors. These results show that the health expenditures made over the years do not provide any added value for liver donors, and role of women on liver donor is significantly dominant.

## 1. Introduction

A liver transplant involves surgery to remove a damaged or diseased liver and replace it by transplanting a healthy liver, either in its entirety or in part, from a donor.^[1]^ The success rate of liver transplant result has grown due to the development of novel immunosuppressants and preservation solutions, enhanced surgical techniques, early disease identification, and effective management of symptoms.^[2]^ However, metabolic disease, which includes hypertension, diabetes mellitus, obesity, dyslipidemia, and hyperuricemia, is a risk factor for cardiovascular disease as well as a frequent side effect following liver transplantation. Immunosuppressive drug adverse effects are closely linked to the development of metabolic illness.^[3,4]^ For the health of the patient after transplantation, the donors must also be healthy. For these reasons, liver transplantation is an important for patients before and after procedure.^[5]^ Following liver transplants, the main causes of death were found to be ischemia trauma to the donor organs and injury from reperfusion to the graft, which resulted in sepsis or liver failure.^[6]^ In order to reduce negative effects of liver related diseases and increase success of liver transplantations, donor numbers become important for both patient and public health perspective. Although the number of donors in liver transplantation is not directly related to the success of the treatment process, research shows that as the number of donors increases, the field will find healthy donors. Da et al^[7]^ reported that 1887 (7.7%) of the 24,500 donors used for liver transplants were positive for the hepatitis C virus by antibody, and 64.4% of these donors also tested positive for the virus by nucleic acid amplification. These striking figures revealed in the research indicate that more donors will be needed to select suitable volunteer donors before organ transplantation.

Basically, an organ may be donated alive or after death. Although not every organ may be donated while alive, living organ donation is possible for the liver.^[2,8–10]^ Becoming a donor for an organ is an important decision, and this decision relates to both the individual and the people around the donor. According to the Euro Health Consumer Index, organ donation rates using deceased donors and organizational strategies for organ donation vary significantly throughout nations with comparable levels of healthcare.^[11]^ It is critical and pressing to ensure the safety of organ donation and transplantation, to direct recipients’ disease prevention, and to maximize the appropriate diagnosis and treatment protocol.^[12]^ To achieve this, the number of organ donors must increase and become widespread. The most common type of transplantation is still the use of deceased donors’ organs.^[13]^ However, for faster and healthier transplantation of organs, the number of living donors must increase as well as the number of deceased donors.

Since the decision to donate an organ affects not only individuals but also their environment, society and culture, the number of donors should be examined in terms of social variables. Although social factors are effective and important in organ donation, sufficient studies have not been found in this field. In addition, there is no consensus in the literature regarding social variables. However, examining social variables on organ donation, which is an important issue for individuals and the healthcare system, can make a positive contribution to the field. This study aimed to reveal longitudinally the effect of community factors on the number of donors for liver transplant, where the number of living donors is important. In this way, it was aimed to reveal which variables affect the number of donors and to make a series of suggestions to increase the number of donors.

## 2. Methods

### 2.1. Model of the research

Relational and descriptive scanning models were used in the study. The objective in this instance was to characterize and assess the connection between the quantity of liver donors and the demographics of the surrounding neighborhood.

### 2.2. Dataset sample

Liver donor data were collected from the USA Health Resources & Services Administration-Organ Procurement and Transplantation Network for 1988 to 2023 time periods.

Dependent variables (the USA Health Resources & Services Administration-Organ Procurement and Transplantation Network):

• Living Liver Donor
• Deceased Liver Donor

Independent variables (the World Bank Country Reports):

• Domestic general government health expenditure (% of GDP)
• Current health expenditure (% of GDP)
• Rural population growth (annual %)
• Population, male (% of total population)
• Age dependency ratio, young (% of working-age population)
• Educational attainment, at least completed lower secondary, population 25+, female (%) (cumulative)
• Employment to population ratio, 15+, total (%) (modeled ILO estimate)
• GDP per capita, PPP (current international $)
• Gini index

### 2.3. Statistical methods

The parameters relating to donors and the community demography were presented using means, standard deviations, and ranges. The normality distribution of the research parameters was assessed using the Kolmogorov–Smirnov test. Nonparametric tests were utilized since all research parameters distributed nonnormally. The association between donor and community demography rates was examined using Spearman rho and year-controlled partial correlation analysis. Regression models’ linearization deviations led to the application of the Generalized Linear Model-Logit (GLM-L) to examine the effects of substantially correlated factors on transplantation.^[14,15]^ With a 95% Confidence Interval and a 0.05 significance threshold, SPSS 25.0 for Windows was utilized.

## 3. Results

Deceased donor number was ranged between 577 and 2048 with 1434.36 ± 351.00 mean value. Living donor number was ranged between 0 and 11 people, and the mean value was 2.00 ± 2.73. Government health expenditure mean was 7.60 ± 1.18% of GDP, and mean current health expenditure in GDP % was 15.58 ± 1.43. Rural population growth percentage was negative showing urbanization with −0.23 ± 0.33 percentage mean. Male percentage mean was 49.25 ± 0.19 with 48.90 to 49.56 range. Age dependency ratio mean was 30.84 ± 1.80. Female education attainment level mean was 94.85 ± 1.21. Employment ratio mean was 60.26 ± 1.89, GDP per capita mean was 44,959.98 ± 14,319.00 and Gini index mean was 40.25 ± 1.03 (Table 1).

**Table 1:** Descriptive statistics of donor numbers and related social parameters (1988–2023).

	Minimum	Maximum	Mean	Std. deviation
Deceased donor, person	577.00	2048.00	1434.36	351.00
Living donor, person	0.00	11.00	2.00	2.73
Government health expenditure (% of GDP)	5.54	10.68	7.60	1.18
Current health expenditure (% of GDP)	12.49	18.82	15.58	1.43
Rural population growth (annual %)	−1.06	0.36	−0.23	0.33
Male percentage (% of total population)	48.90	49.56	49.25	0.19
Age dependency (% of working-age population)	27.67	33.30	30.84	1.80
Female education attainment, female (%)	91.36	96.55	94.85	1.21
Employment to population ratio, 15+, total (%) (modeled ILO estimate)	56.38	63.30	60.26	1.89
GDP per capita, PPP (current international $)	23,888.60	76,398.59	44,959.98	14,319.00
Gini index	37.70	41.50	40.25	1.03

**Figure 1.**
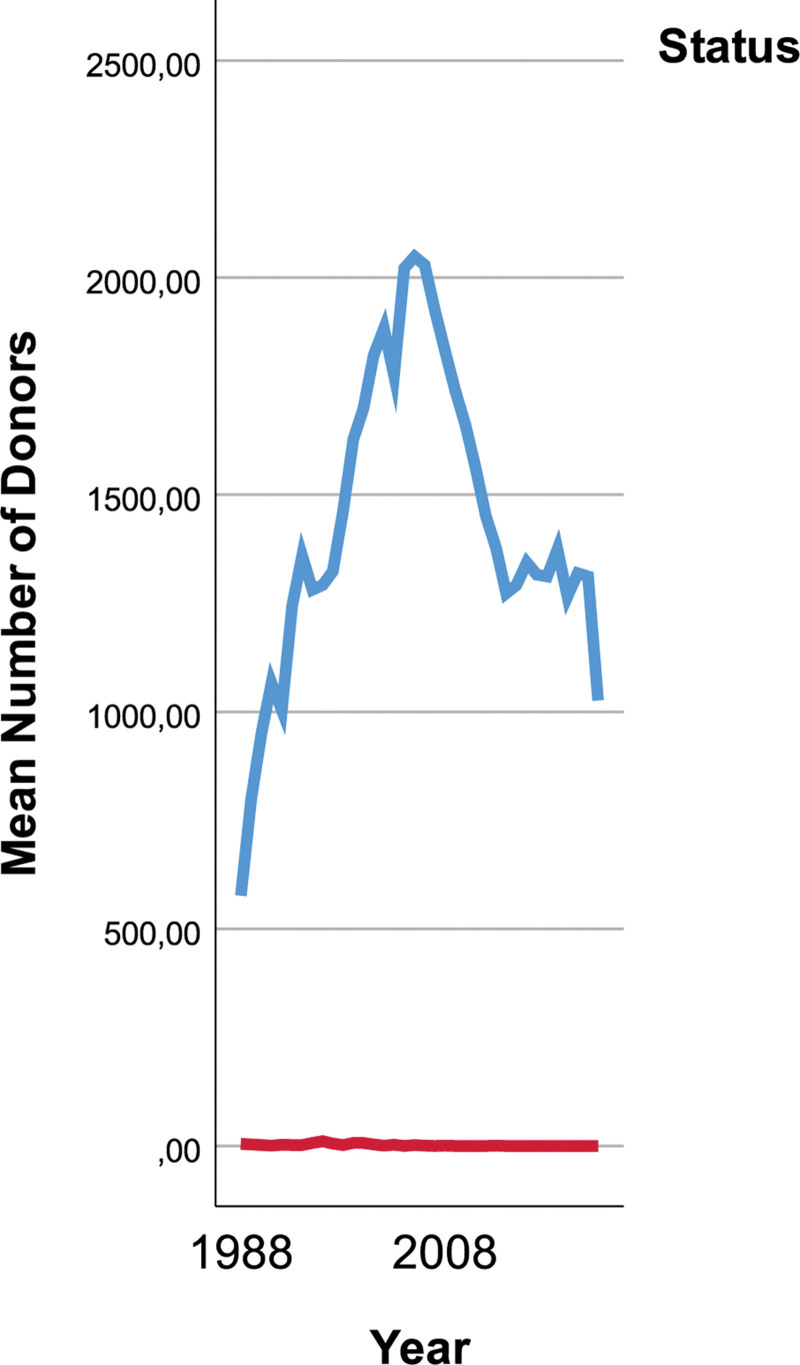
Yearly change in numbers of living and deceased liver donors.

Deceased liver donor numbers were increasing trend until 2006, and decreased after 2007. Living liver donor numbers were very low, and changes were insignificant within the time period (Fig. 1).

According to Spearman rho correlation analysis, deceased donor numbers were significantly and negatively correlated with government health expenditure (*r* = −0.816; *P* < .01), current health expenditure (*r* = −0.768; *P* < .01), female education attainment (*r* = −0.804; *P* < .01) and Gini index (*r* = 0.434; *P* < .05). Living donor numbers were significantly and negatively correlated with government health expenditure (*r* = −0.731; *P* < .01), current health expenditure (*r* = −0.781; *P* < .01), male percentage (*r* = −0.786; *P* < .01), female education attainment (*r* = −0.640; *P* < .05), employment (*r* = 0.751; *P* < .01), GDP (*r* = −0.792; *P* < .01) and Gini index (*r* = −0.486; *P* < .01). Living donor numbers were significantly and positively correlated with age dependency (*r* = 0.815; *P* < .01) (Table 2).

**Table 2:** Spearman rho correlation analysis between donor numbers and community factors.

	Deceased donor, person		Living donor, person	
	*r*	*P*	*r*	*P*
Government health expenditure (% of GDP)	−0.816**	.000	−0.731**	.000
Current health expenditure (% of GDP)	−0.768**	.000	−0.781**	.000
Rural population growth (annual %)	0.229	.186	0.050	.777
Male percentage (% of total population)	0.258	.134	−0.786**	.000
Age dependency (% of working-age population)	−0.139	.426	0.815**	.000
Female education attainment, female (%)	−0.804**	.000	−0.640*	.010
Employment to population ratio, 15+, total (%) (modeled ILO estimate)	0.344	.054	0.751**	.000
GDP per capita, PPP (current international $)	0.101	.577	−0.792**	.000
Gini index	0.434*	.010	−0.486**	.004

* *P* < .05.

** *P* < .01.

Although correlations between deceased and living liver donor numbers and community factors were significant in Spearman rho correlation analysis, most of year-controlled partial correlation analysis results were statistically insignificant (*P* > .05). For deceased donor, only employment (*r* = 0.471; *P* < .01) and Gini (*r* = 0.439; *P* < .05) were significantly correlated. For living donor, only employment was significantly correlated (*r* = 0.375; *P* < .05) (Table 3).

**Table 3:** Year-controlled partial correlation analysis between donor numbers and community factors.

	Deceased donor, person		Living donor, person	
	*r*	*P*	*r*	*P*
Government health expenditure (% of GDP)	0.385	.194	−0.170	.578
Current health expenditure (% of GDP)	0.301	.318	−0.287	.342
Rural population growth (annual %)	0.361	.226	0.057	.853
Male percentage (% of total population)	0.487	.091	0.299	.321
Age dependency (% of working-age population)	0.253	.404	0.082	.790
Female education attainment, female (%)	0.332	.267	−0.022	.944
Employment to population ratio, 15+, total (%) (modeled ILO estimate)	0.471**	.009	0.375*	.041
GDP per capita, PPP (current international $)	0.019	.921	−0.036	.849
Gini index	0.439*	.015	0.163	.389

* *P* < .05

** *P* < .01

Generalized Linear Model-Logit (GLM-L) results showed that effect of female education attainment had significant contribution on deceased liver donor (*B* = −3290.605; *P* < .01). Effects of significantly correlated community factors on living liver donor numbers were found to be statistically insignificant (*P* > .05) (Table 4).

**Table 4:** Generalized Linear Model (Logit) for effects of related factors on deceased and living liver donor numbers

Parameter	*B*	Std. error	95% Wald confidence interval		Hypothesis test		
			Lower	Upper	Wald Chi-square	df	*P*
*Deceased donor*							
(Intercept)	394,980.919	113,710.4269	172,110.332	617,860.507	120.065	1	.001
Government health expenditure (% of GDP)	2390.066	2580.6928	−2670.962	7460.095	0.854	1	.355
Current health expenditure (% of GDP)	−2550.548	2320.1300	−7100.515	1990.418	10.212	1	.271
Female education attainment, female (%)	−3290.605	1500.7079	−6240.987	−340.223	40.783	1	.029
Gini index	−1060.981	850.9570	−2750.453	610.492	10.549	1	.213
(Scale)	114,620.554	43,320.4381	54,640.588	240,430.923			
*Living donor*							
(Intercept)	4590.569	4260.5188	−3760.392	12,950.531	10.161	1	.281
Government health expenditure (% of GDP)	20.375	10.3522	−0.275	50.025	30.086	1	.079
Current health expenditure, (% of GDP)	−20.562	10.3773	−50.261	0.138	30.459	1	.063
Male percentage (% of total population)	−30.470	90.0150	−210.139	140.199	0.148	1	.700
Age dependency (% of working-age population)	−10.737	20.0534	−50.761	20.288	0.715	1	.398
Female education attainment, female (%)	−20.183	10.4206	−40.968	0.601	20.362	1	.124
Employment to population ratio, 15+, total (%) (modeled ILO estimate)	0.168	0.2063	−0.237	0.572	0.660	1	.417
GDP per capita, PPP (current international $)	0.000	0.0003	0.000	0.001	0.180	1	.671
Gini index	−0.577	0.4927	−10.542	0.389	10.369	1	.242
Scale	0.167	0.0633	0.080	0.351			

## 4. Discussion

The liver is an important organ that plays an active role in many metabolic processes of the body. Therefore, liver diseases are important both in terms of metabolic diseases^[16,17]^ and chronic and malignant diseases.^[18]^ For this reason, it is important patients to have urgent and safety transplant liver in order to have a healthy life.^[19]^

In studies on liver transplantation, it has been reported that being a deceased donor is generally common and the number of living donors is less.^[13]^ A similar situation existed in our study, and the deceased donor average (1434.36 ± 351.00) was significantly higher than the living donor average (2.00 ± 2.73). These findings we obtained were compatible with the literature. However, organ transplantation must be performed urgently in liver disease^[12]^ and due to the high disease rates among donors, the number of donors suitable for transplantation decreases due to diseases.^[7]^ Therefore, it is beneficial for health institutions and public health practitioners, as well as in the medical field, to take measures that will encourage organ donation, increase their numbers and increase donor rates.

Deciding to donate an organ also represents a psychological and cultural process, as it is associated with thinking about death.^[20–22]^ Being an organ donor is actually a concept that may be evaluated through individuals’ empathy, social awareness and health literacy levels.^[23,24]^ Therefore, examining the effects of social and community variables on the donor is of great importance in understanding and managing the tendency towards organ donation in society.

The research results showed that the government’s health expenditures were around 7.60% on average, and current health expenditures had an average value of 15.58%. This shows that healthcare expenditures in the US healthcare system are mostly made privately and the government’s contribution is low. It is possible that this situation may have an impact on the number of liver donations. According to the correlation analysis results we obtained in the study, the effects of both the government’s health expenditures and total health expenditures on the number of donors were statistically significant and negative. In other words, as health expenditures increased, the number of donors decreased. In fact, what is expected is that health expenditures will make a positive contribution to all areas of health and the number of donors. However, the negative correlation indicates that health expenditures are mostly related to treatment processes and acute health conditions.

Although increasing the number of living donors is important for liver transplantation, it seems that the number of deceased donors is higher. According to the results of the correlation analysis, the relationship between the number of deceased donors and the government and current health expenditures was significant, and the rate of women’s participation in education also had a decreasing effect on the number of deceased donors. For the living donor, the relationships with government and current health expenditures, gender structure of the population, age dependency ratio and female education participation levels were significant. While there was a statistically significant and positive relationship only between age dependency ratio and living donor, the relationships between all other community factors and living donor were negatively significant. These indicators point out that as the level of health and social demographics increases, the level of donor status decreases, revealing that there is a serious problem in the health literacy system. According to the results of year-controlled partial correlation analysis, the relationships between the numbers of deceased and living liver donors and all research variables were found to be insignificant. This shows that there has been no development or statistically significant progress regarding liver donors over time. Among the important parameters among social factors, employment, economic situation and income distribution justice are also included.^[25–27]^ While employment is an indicator of the social and economic status of individuals, income indicates purchasing power and social status. Income distribution justice is an indicator of the bond of individuals within a society to that society.^[28,29]^ The results we obtained in our research showed that there is a significant relationship between the number of living donors and the Gini coefficient among these variables.

The results of the GLM-L analysis conducted to reveal the level of impact revealed that women’s participation in education was the only effective parameter on the number of deceased liver donors. Although according to the correlation analysis results, there was a significant relationship between the number of deceased liver donors and government health expenditures and current health expenditures, this relationship did not turn into an effect in multivariate analysis. Although there may be many reasons for this, it can be interpreted that women’s participation in education and therefore in the employment structure causes additional health burden and less social awareness. The effect of factors whose correlation analysis results were significant on the number of living donors was not significant. It is possible to interpret this situation as community factors not having a positive effect on individuals’ ability to become liver donors. Although social variables are effective with more than 1 parameter in the correlation analysis performed as a relational screening analysis, multivariate analysis and regression results showed that female education was the most significant parameter among all the variables. As a result, although these findings are based on scientifically valid and reliable data from 1988 to the present, it is possible to state that the changing patriarchal structure and the woman’s role as a mother have an impact on this perspective.

## 5. Limitations of the study

The most important limitation of the study is that the information about the number of donors and the current status of the donors is quite limited. Despite this, the USA Health Resources & Services Administration-Organ Procurement and Transplantation Network has developed a very comprehensive and successful database and updates the system every day. Therefore, it is possible to attribute this limitation of the research to the fact that it is also a pioneering study in the field.

Another important limitation of the research is that these data are only for the USA, so there is not enough data to apply more comprehensive and advanced analysis methods such as panel data analysis for the situation worldwide. Although donor data is comprehensive in The USA data, date ranges and data sharing with the World Bank are more limited in the World Bank data.

### 5.1. Contribution to literature

The most important contribution of the research to the literature is that there has been no similar study before and therefore it is a pioneering study in the field. Generally, liver transplant and donor studies have either been conducted on very technical and clinical data, or the subject has been addressed in a lower context using very limited samples. In this respect, the research is important as it examines the effect of macro indicators on liver donor numbers.

Another important contribution of the research to the field is that it is approached pragmatically and therefore aims to provide benefits both in the field and in practice. By identifying and revealing the factors affecting the number of donors, it is possible to increase the number of donors by conducting studies on the impact levels of these factors. In this way, it is possible to increase the level of knowledge in the field, both for patients waiting for liver transplantation, for public health, and with the increasing number of donors. In this respect, the research contributes to the field.

## 6. Conclusion

The research findings reveal that, among community factors, especially women’s participation in education has a statistically significant effect on liver donors. When the results of correlation analysis and regression analysis are evaluated together, it shows that the health expenditures made over the years do not provide any added value for liver donors. It reveals that more studies on this subject and a focus on liver donors, especially to increase the number of living donors, are necessary.

## Acknowledgments

We thank Kadir Yilmaz with his valuable statistics support.

## Author contributions

**Data curation:** Ender Anilir.

**Formal analysis:** Ender Anilir.

**Writing – original draft:** Ender Anilir.

**Writing – review & editing:** Ender Anilir.
